# Two new species of braconid wasps (Hymenoptera, Braconidae) from India

**DOI:** 10.3897/zookeys.889.36436

**Published:** 2019-11-14

**Authors:** Zubair Ahmad, Hamed A. Ghramh, Anjum Ansari

**Affiliations:** 1 Research Center for Advanced Materials Science (RCAMS), King Khalid University, 9004, Abha 61413, Saudi Arabia; 2 Unit of Bee Research and Honey Production, Faculty of Science, King Khalid University, P.O. Box 9004, Abha 61413, Saudi Arabia; 3 Biology Department, Faculty of Science, King Khalid University, P.O. Box 9004, Abha 61413, Saudi Arabia; 4 Biology Department, Faculty of Sciences and Arts, Dhahran Al Janoub, King Khalid University, Saudi Arabia; 5 Department of Zoology, Aligarh Muslim University, Aligarh, 202002, UP., India

**Keywords:** Braconidae, coleopteran leafminer host, *
Corchorus
*, *
Pambolus
*, *
Parachremylus
*, taxonomy, *
Trachys
*

## Abstract

Two new species viz., Pambolus (Phaenodus) shujai**sp. nov.**, and *Parachremylus trachysi***sp. nov.**, of braconid wasps are described as new to science. *Parachremylus trachysi***sp. nov.**, is reared from larvae of the leaf miner *Trachys* sp. (Coleoptera, Buprestidae) on *Corchorus* sp. (Wild Jute Plant). A new species of *Pambolus* Haliday along with two known species is also recorded. A key to the Indian species of *Pambolus* is also provided. Diagnoses with morphological characters and illustrations are provided.

## Introduction

Braconid wasps (Hymenoptera, Braconidae) represent an important insect group of natural parasitoids which have an efficient role in biological control programs (Matthews 1974, Shaw 1995). Braconids are speciose with 19,801 described species belonging to 1071 genera, which represent nearly 20% of the total hymenopteran diversity worldwide (van Achterberg 2014, Yu et al. 2016). Braconids are distributed in all zoogeographical regions (Wahl and Sharkey 1993) and play an ecological role as regulators of other insect groups. The majority of the species are parasitoids especially upon the larval stages of insect pests in the various orders including Hemiptera, Coleoptera, Diptera, Hymenoptera, and Lepidoptera (Shaw and Huddleston 1991, Whitfield and Wharton 1997).

In the present work, two new braconid species, one each from the genus *Pambolus* Haliday and the genus *Parachremylus* Foerster are described as new to science. Taxonomically the genus *Pambolus* was included either in the subfamily Hormiinae Foerster, 1862 s. l. (Whitfield and Wharton 1997) or in a separate subfamily Pambolinae Marshall, 1885 (van Achterberg 1995, Braet and van Achterberg 2003, van Achterberg and Braet 2004). The subsequent classification is most popular among the recent workers (Martínez et al. 2012) as it is based upon a natural group as revealed by a previous phylogenetic study of the cyclostome subfamilies of Braconidae (Zaldívar-Riverón et al. 2006). Presently *Pambolus* is subdivided into two subgenera *Pambolus* (females always brachypterous or micropterous, and males always macropterous) and *Phaenodus* (females always macropterous, and males usually macropterous) (Belokobylskij and Kula 2012). *Phaenodus* is often treated as a separate genus (see Belokobylskij 1986, Belokobylskij 1988, Belokobylskij 1992b, Papp 1996, 2000, Belokobylskij 1998, 1999, Braet and van Achterberg 2003). The genus *Pambolus* is cosmopolitan in distribution and is particularly diverse in the Neotropics (Whitfield and Wharton 1997). Of the 43 species worldwide, six are reported from the Oriental region (Yu et al. 2016) of which three species, viz., P. (Phaenodus) ignarus Papp, P. (Ph.) topali Papp and P. (Ph.) ruficeps Belokobylskij are reported from India (Papp 1996). All species known from India, including the new species described herein, belong to the subgenus Phaenodus. A key to the Indian species of the genus *Pambolus* is also provided in this paper.

The systematic position of the genus *Parachremylus* Granger is disputed, either included in the subfamily Exothecinae (tribe Avgini Belokobylskij, 1993) or more traditionally in the subfamily Hormiinae (Wharton 1993). The genus *Parachremylus* is restricted to the Old World tropics and is represented by only four species viz., *P. litchi* Belokobylskij & Maeto, *P. oblongus* (Papp), *P. seyrigi* Granger and *P. temporalis* Belokobylskij (Papp 1996, Papp 1997, Belokobylskij and Maeto 2006, Yu et al. 2016). In the present work, one new species of *Parachremylus* is described from the leafmining larvae of *Trachys* sp., on *Chorchorus* sp., a wild jute plant in India. This is the first record of this genus reared from coleopteran leafminer hosts. Other species of this genus viz., *P. litchi* (Belokobylskij and Maeto 2006) were reared from larvae of *Conopomorpha sinensis* and *C. litchiella* (Lepidoptera: Gracillariidae).

## Materials and methods

The specimens were collected from northern Uttar Pradesh in order to study the biodiversity and conservation of parasitoid wasps in the northern region of India. The subfamily keys of van Achterberg (1993) and Wharton et al. (1997) and generic keys of Belokobylskij (1993) and Wharton (1993) were used for the identification. Descriptions by Belokobylskij (1992a) and Papp (1996) were used for *Pambolus*, and descriptions by Belokobylskij and Maeto (2006) were used for determining *Parachremylus* species. We followed Sharkey and Wharton (1997) for terminology of various body parts and wing venation and Eady (1968) for the terminology of micro-sculpture. The following abbreviations are used in the text: **OOL** – ocello-ocular line (distance from the outer edge of a lateral ocellus to the compound eye); **POL** – post-ocellar line (distance between the inner edges of the two lateral ocelli); **AOL** – anterior-ocellar line (distance between the inner edges of anterior and lateral ocellus); **ØOD** – diameter of an ocellus; **T1** – First metasomal tergite; **F1** – First antennal flagellomere. All descriptions, measurements, and photographs of wings and body parts were made under a Zeiss Discovery V20 stereo zoom microscope while scanning electron microscope (SEM) photomicrographs were taken using a LEO 435VP SEM. The specimens have been deposited in the Insect Collection section of the Department of Zoology, Aligarh Muslim University, Aligarh, India (ZDAMU).

## Results

### Taxonomy

#### Genus *Pambolus* Haliday, 1836

##### Key to the Indian species of Pambolus (Phaenodus) Haliday (females)

**Table:** 

1	Female length 3–4.2 mm; notauli distinct throughout, deep, crenulated; antennae about 1.5–1.7 × as long a body; propodeal spines as long as second and third tarsomere of hind tarsus; face and vertex sculptured usually rugose to rugulose	**2**
–	Female length at most up to 2.8 mm; notauli indistinct anteriorly, rather prominent posteriorly; antennae about 2.0 × as long as body; propodeal spines short, half as long as third tarsomere of hind tarsus; face polished, vertex smooth to finely granulate	**3**
2	Eyes about 2.0 × as long as temple in dorsal view, latter rounded; hind femur 4.6–5.0 × as long as broad medially; antennae with 27–30 segments. T1 as long as broad apically; vertex, and occiput rather transversely rugulo-rugose	**P. (Ph.) topali Papp**
–	Eyes 2.7–3.0 × as long as temple in dorsal view, latter receded; hind femur 3.5–3.8 × as long as broad medially; antennae with 33–40 segments. T1 1.1–1.3 × as long as broad apically; vertex and, occiput coriaceous to rugulose	**P. (Ph.) ruficeps Belokobylskij**
3	Antennae yellowish brown; malar space 2.0–2.5 × basal width of mandible; pterostigma 4.0–5.0 × as long as wide; propodeal spines located anterior to middle of propodeum; antennae 26 segmented	**P. (Ph.) ignarus Papp**
–	Antennae with F17–F29 creamish-white; malar space 3.5 × basal width of mandible; pterostigma 3.0 × as long as wide; propodeal spines located in the middle of the propodeum; antennae 29 segmented	**P. (Ph.) shujai Ahmad, sp. nov.**


##### Pambolus (Phaenodus) ignarus

Taxon classificationAnimaliaHymenopteraBraconidae

Papp

F06045B2-20E0-5BCC-AF1A-D0FFE5C2B1AB

Figs 1–3

Pambolus (Phaenodus) ignarus Papp, 1996: 46; Yu et al. 2016.

###### Material examined.

2 females, “INDIA: Uttar Pradesh, Aurriya, 23.IX.03; coll. M Shamim (ZDAMU)”. 1♀, “INDIA: Uttar Pradesh, Aligarh, 09.X.01; Coll. Zubair Ahmad (ZDAMU)”.

###### Remarks.

Pambolus (Ph.) ignarus is known among all the Oriental species by its smaller size and almost absence of notauli, which is hardly impressed on the anterior part of mesoscutum and without any crenulation. A brief diagnosis is as follows: body length 1.8 mm long; forewing length 1.9 mm; ground color of head brownish yellow; meso- and metasoma rusty brown; antennae unicolor (yellowish brown); head in dorsal view less transverse 1.7 × as broad as long; eye 4.0 × as long as temple; antenna nearly 2.0 × as long as body and with 25 segments; F1 5.0 × as long as broad apically; face polished; notauli hardly distinct on disc of mesonotum; mesonotum finely granulose; propodeal spines short, as long as half of third tarsomere of hind tarsus; forewing vein r arising beyond middle of pterostigma; ovipositor sheath as long as hind basitarsus.

###### Host.

Unknown.

###### Distribution.

India: Karnataka, Uttar Pradesh (Papp 1996).

**Figures 1–3.**
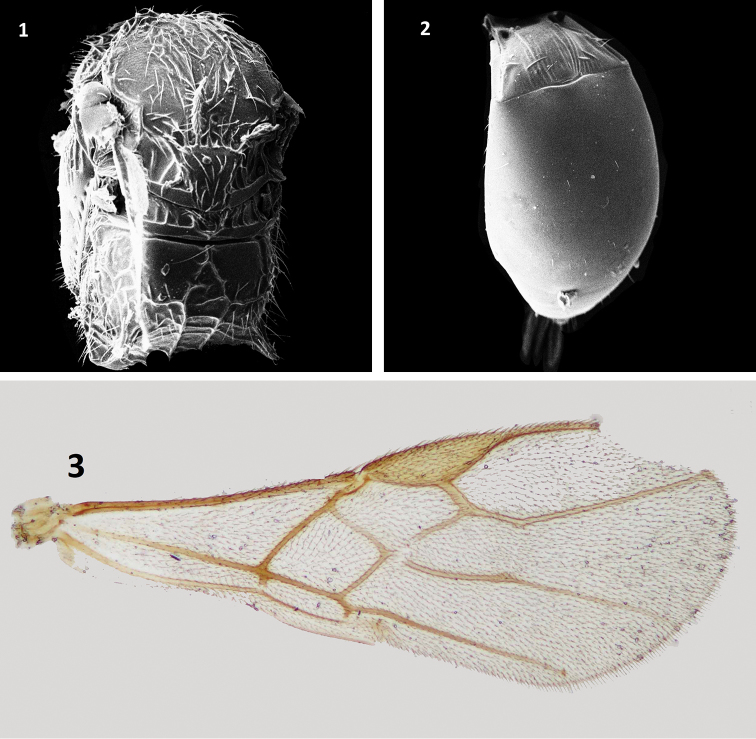
Pambolus (Ph.) ignarus Papp **1** mesosoma, dorsal view **2** metasoma, dorsal view **3** forewing.

##### Pambolus (Phaenodus) shujai

Taxon classificationAnimaliaHymenopteraBraconidae

Ahmad sp. nov.

F9F4D8BB-FE75-5BE0-BCC2-3CEB2F475469

http://zoobank.org/D6EE6537-36D1-4F99-B972-71C77A6D9243

Figs 8 , 9

###### Material examined.

**Holotype**, female, “INDIA: Uttar Pradesh, Etawah, 13.IV.02; coll. M Shamim (ZDAMU)”. **Paratypes**, 3 females with same data as holotype.

###### Diagnosis.

The new species *Pambolus shujai* Ahmad sp. nov., is closely related to *Pambolus ignarus* Papp. However, it differs from *P. ignarus* in having (1) antennae with F17–F29 creamish-white (antennae yellowish brown in *P. ignarus*); (2) POL: ØOD: OOL = 2:1.5:6 (POL: ØOD: OOL = 2: 2: 5 in *P. ignarus*); (3) antennae as long as body (antennae 2.0 × as long as body length in *P. ignarus*); (4) propodeal spines present at the middle of propodeum, length of spine 0.4 × as long as fore basitarsus, 0.3 × the distance between them (pair of lateral spines present at one-third below the middle of propodeum; length of spines as long as fore basitarsus, 0.5 × the distance between them length in *P. ignarus*); (5) hind basitarsus 1.2 × as long as tarsal segment 2–4 combined (hind basitarsus 0.7 × as long as tarsal segment 2–4 combined in *P. ignarus*); (6) forewings distinctly shorter than the body (forewings distinctly longer than the body *P. ignarus*). (7) pterostigma 3.0 × as long as wide (pterostigma 5 × as long as wide *P. ignarus*). The new species also differs from other Indian species, *P. ruficeps* Belokobylskij, by having (1) vertex finely granulates around ocelli, smooth elsewhere (rugulose in *P. ruficeps*); (2) forewing veins r, 2RS, 3RSa thickened (forewing veins r, 2RS, 3RSa normal in *P. ruficeps*); (3) antennae as long as body (antennae 1.5–1.7 × as long as body length in *P. ruficeps*); (4) face smooth (face reticulate rugulose in *P. ruficeps*).

###### Description.

Female, body length: 2.8 mm; forewing: 2.3 mm.

***Head.*** Antennae 29 segmented, almost as long as body; scape 1.6 × as long as wide, F1 3.5–4.0 × as long as wide, head transverse, ca. 2.0 × as wide as long in dorsal view, temple smooth, distinctly widening ventrally, widest distance from eyes 2.0 × as long as eye dorsally; AOL: POL: ØOD: OOL =1:2:1.5:6; malar space 3.5 × basal width of mandible; face sparsely setose, smooth, 1.3 × as wide as long; clypeus smooth, distinctly separated from face, slightly convex, 3.5 × as wide as long; hypoclypeal depression elliptical almost 3.0 × as wide as long medially; frons strigose with few setae; vertex finely granulate around ocelli, rest smooth.

***Mesosoma.*** Mesosoma 1.8 × as long as wide in dorsal view, 2.0 × as long as high in lateral view; pronotum small and sparsely setose; median and lateral lobes of mesoscutum granulate, sparsely setose; notauli poorly developed anteriorly, distinct posteriorly with some longitudinal carinae; scutellar sulcus deep and broad with four crenulae, 0.6–0.7 × as long as scutellum, scutellum polished with fine setae laterally and posteriorly, side of scutellum concave with longitudinal striations, metanotum almost at the same level, longitudinally striated, sparsely setose; propodeum with strong areolation, areola elongate, with a pair of spines, present at the middle of propodeum; propodeal spine 0.4 × as long as fore basitarsus and 0.3 × the distance between them; propleuron anteriorly granulate, posteriorly rugose, sparsely setose; mesopleuron anteriorly rugose, otherwise smooth and polished; episternal scrobe deep and isolated, crenulate at the margins.

***Wings.*** Forewing 2.7 × as long as wide, 0.8–0.9 × as long as body, 2.7 × as long as hind tibia; pterostigma 3.0 × as long as wide, 0.8 × length of R1a, r arising from its middle; r about as long as width of pterostigma; second marginal cell of moderate size; 3RSa 0.9 × as long as r, 0.5 × 2RS, 0.25 × 3RSb; r-m 0.7 × 3RSa; CU1b arising from the middle of brachial cell; marginal cell somewhat short about 2.25 × as wide as high, 3RSb straight and falls much before the tip of wing; (RS+M)a slightly curved; hind wing 4.0 × as long as wide; M+CU 0.6 × 1M.

***Legs.*** Hind leg setose, hind femur 4.0 × as long as broad, hind tarsus 0.9 × as long as hind tibia, hind basitarsus 1.2 × as long as tarsal segment 2–4 combined.

***Metasoma.*** Metasoma as long as head and mesosoma combined in dorsal view, 2.0 × as long as wide; T1 longitudinally striated, strongly broadening posteriorly; 2 × as long as broad basally, spiracles present a little basally from middle; further tergites polished; ovipositor sheath in lateral view 1.1 × as long as tarsomere 2–4 combined; ovipositor short, straight and pointed.

***Color.*** Head brownish yellow with dark brown patches; eyes, stemmaticum, propleuron, mesopleuron, propodeum black; mesonotum, metanotum, legs brownish; metasoma reddish brown; ovipositor sheath brownish; mandibles yellowish brown; tip of mandible, claws, antennal segment F1–F16 dark brown; F17–F29 creamish-white; ocelli transparent; wings hyaline, pterostigma yellowish brown, veins yellowish brown and thickened.

###### Male.

Unknown.

###### Host.

Unknown.

###### Distribution.

India (Uttar Pradesh).

###### Etymology.

The species is named after Dr Shujauddin for his valuable contributions to the taxonomy of Indian Braconidae.

##### Pambolus (Phaenodus) ruficeps

Taxon classificationAnimaliaHymenopteraBraconidae

Belokobylskij

34B0F990-7D5B-5D34-99B8-3A5961FDE82C

Figs 4–7

Pambolus (Phaenodus) ruficeps Belokobylskij, 1988: 27 (Taiwan); Belokobylskij 1990: 128 (Malaysia) Belokobylskij 1992a, 167 (Vietnam); Papp 1996: 50 (India)

###### Material examined.

2 females, “INDIA: Uttar Pradesh, Etawah, 13.IV.02; coll. M Shamim (ZDAMU)”.

###### Diagnosis.

Pambolus (Ph.) ruficeps is quite unlike all Oriental species of the genus *Pambolus* due to the presence of a heavily sculptured head. A brief diagnosis of P. (Ph.) ruficeps follows: body length 3.0–4.2 mm long, forewing length 3.0 mm long; eye 2.7–3.0 × as long as temple in dorsal view, latter receded; antenna with 33–40 segmented, 1.5–1.7 × as long as body; vertex, occiput coriaceous to rugulose; face reticulate rugulose; notauli distinct and crenulated; vein r, 2RS, 3RSa normal; propodeal spine as long as second or third tarsomere of hind tarsus; hind femur 3.5–3.8 × as long as broad medially; F1 1.1–1.3 × as broad behind as long medially.

###### Host.

Unknown.

###### Distribution.

India: Jammu and Kashmir, Orissa and Uttar Pradesh; Malaysia, Taiwan, Vietnam (Papp 1996).

**Figures 4–7.**
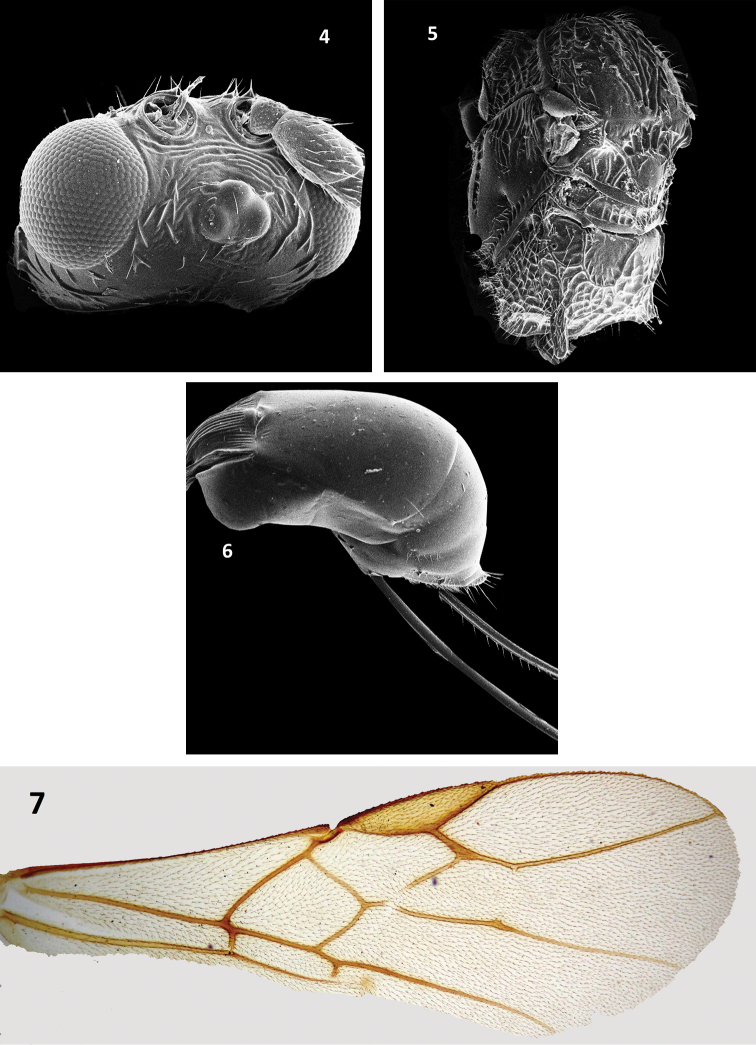
Pambolus (Ph.) ruficeps Belokobylskij **4** head, dorsal view **5** mesosoma, dorso-lateral view **6** metasoma, lateral view **7** forewing.

**Figures 8, 9.**
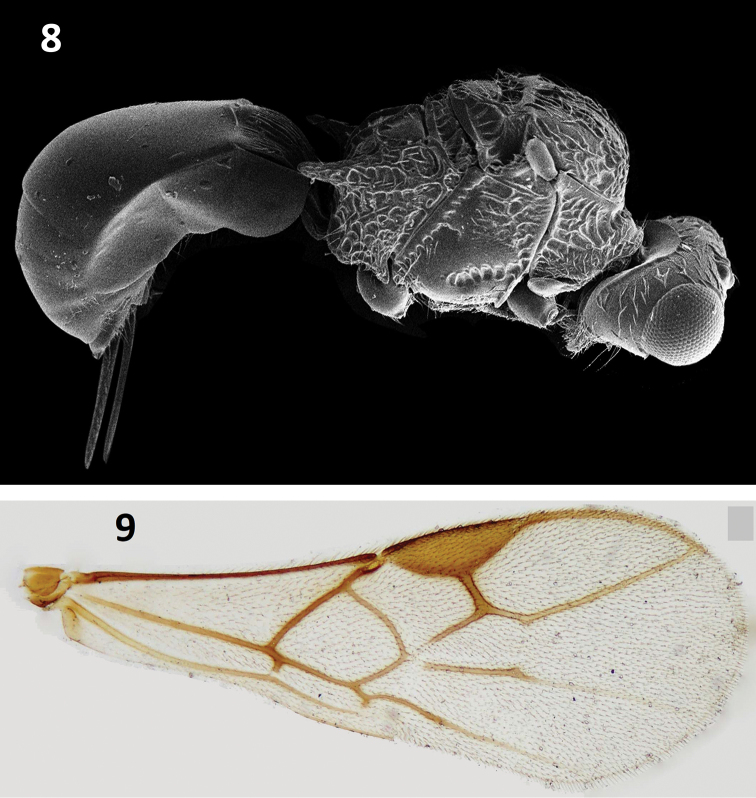
Pambolus (Ph.) shujai Ahmad sp. nov. **8** habitus, lateral view **9** forewing.

#### *Parachremylus* Granger, 1949

##### Parachremylus trachysi

Taxon classificationAnimaliaHymenopteraBraconidae

Ahmad sp. nov.

FA9BA224-6D9B-5717-B30D-45515B9C2143

http://zoobank.org/6837B17F-77C7-48C1-A5A6-B11EAEFA9C69

Figs 10–13

###### Material examined.

**Holotype**, female, “INDIA: Uttar Pradesh, Aligarh, 10.VIII.2005; ex *Trachys* sp. on *Corchorus* sp., Coll. Zubair Ahmad (ZDAMU)”. **Paratypes**, 3 females and 2 males; with same data as holotype.

###### Diagnosis.

Following the key to world species of *Parachremylus* by Belokobylskij and Maeto (2006), *Parachremylus trachysi* sp. nov., runs near to another Indian species viz., *P. oblongus* (Papp). The new species shares similarities with *P. oblongus* in having the presence of longer temple; transverse diameter of eye 4.0 × as long as temple length in dorsal view; malar space 1.2 × basal width of mandible; mesopleuron smooth in upper half, striation partly present in subalar depression only; 1–4 segments of hind tarsomere with narrow and partly indistinct flanges and shallow field of hind half of mesonotum with a medio-longitudinal carinae running up to middle of mesoscutum. However, the new species can easily be distinguished from *P. oblongus* by having the head and mesosoma densely setose (head and mesosoma sparsely setose in *P. oblongus*); median longitudinal carina of metasoma reaching up to almost T3 (median longitudinal carina of metasoma reaching up to almost T2 in *P. oblongus*); pterostigma 3.5 × as long as high (pterostigma 5.0 × as long as high in *P. oblongus*); malar space 1.2 × as long as basal width of mandible (malar space 0.75 × as long as basal width of mandible in *P. oblongus*); scutellar sulcus with three crenulae (scutellar sulcus with five crenulae in *P. oblongus*); forewing veins 2RS and 3RSa 3.5 × as long r (forewing veins 2RS and 3RSa 2.7 × as long r in *P. oblongus*)

###### Description.

Female, body length: 2.1 mm; forewing: 2.0 mm.

***Head.*** Antennae 27 segmented, about as long as body length, F1– F2 3–3.3 × as long as apical width, except the apical segment which is 4.5 × as long as wide; head transverse, 1.5–1.7 × as wide as long in dorsal view and 1.2 × as high as long in lateral view; eyes in lateral view 1.2 × as high as wide, 4.0 × as long as temple, inner margin of eyes not parallel; temple granulate, sparsely setose, its widest part behind eye 0.25 × as long as width of eye; occipital carina dorsally slightly curved towards ocelli, rather widely interrupted medially; occiput with uniform transverse striations; AOL: POL: OOL: ØOD = 2:1:3:2; face polished, laterally shagreened, sparsely setose, 1.5 × as wide as long medially; clypeus 1.6 × as wide as long; vertex granulate, sparsely setose; malar space 0.4 × as long as eye and 1.2 × as long as basal width of mandible;

***Mesosoma.*** Mesosoma 1.5 × as long as wide in dorsal view , 1.6 × as long as high in lateral view; pronotum small, indistinct; median and lateral lobes of mesoscutum granulate, sparsely setose; notauli broad anteriorly, shallow posteriorly, shallow field of hind half of mesonotum with a medio-longitudinal carinae running up to middle of mesoscutum; scutellar sulcus deep and 3.5 × as wide as long with three crenulae; scutellum indistinctly granulate, rather densely setose; propodeal areola with a median longitudinal carina anteriorly, two transverse carina inside, lateral areola with longitudinal striations anteriorly and inside with transverse ruguae; propleuron smooth; mesopleuron smooth and polished except few striation at subalar depression.

***Wings.*** Forewing 2.5 × as long as wide, as long as body; pterostigma 3.0 × as long as wide, issuing r from its middle; length of marginal cell along R1a about 1.2 × as long as pterostigma; 2RS and 3RSa 3.5 × as long r respectively; hind wing vein M+CU 0.8 × 1M; hind femur 3.4–3.6 × as long as broad medially; hind tibia 1.2 × as long as hind tarsus; hind basitarsus 0.6 × as long as tarsomere 2–4 combined.

***Metasoma.*** Metasoma dorsally sub-sclerotized, 1.0 × as long as head and mesosoma combined, T1 evenly and distinctly broadening posteriorly, T2 2.1 × as broad as long, 1. 5 × as long as T3; T3 3.0 × as broad as long; T4 4.0 ×, as wide as long medially; basal pair of carinae of T1 meeting at its basal one-third and continuing a medio-longitudinal carinae up to apical end of T3; all tergites sub- sclerotized; ovipositor sheaths setose and blunt, in lateral view 1.2 × as long as hind basitarsus.

***Color.*** Vertex yellow with brown marking, mandibles, scutellar sulcus, metanotum creamish; tip of mandible, mesoscutum, claws, dark brown; antennae brown; ocelli transparent; eyes, stemmaticum black; wings hyaline, pterostigma pale yellow, veins brown; scutellum, propodeum, legs yellow.

###### Male.

Same as ♀ except body size (2 mm).

###### Host.

*Trachys* sp. on *Corchorus* sp. (Wild Jute).

###### Distribution.

India (Uttar Pradesh).

###### Etymology.

The species name is derived from the name of the genus of the host insect.

**Figures 10–13.**
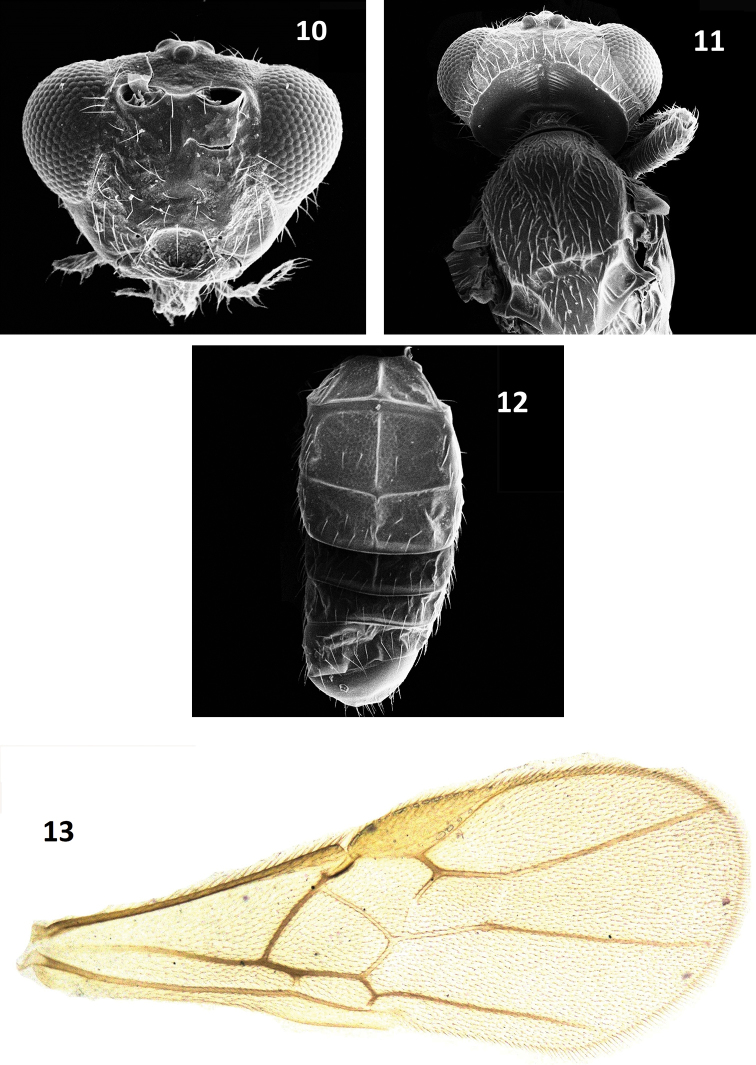
*Parachremylus trachysi* Ahmad, sp. nov. **10** head, anterior view **11** mesosoma, dorsal view **12** metasoma, dorsal view **13** forewing.

## Supplementary Material

XML Treatment for Pambolus (Phaenodus) ignarus

XML Treatment for Pambolus (Phaenodus) shujai

XML Treatment for Pambolus (Phaenodus) ruficeps

XML Treatment for Parachremylus trachysi
